# Biosorption of Aspirin, Salicylic Acid, Ketoprofen, and Naproxen in Aqueous Solution by Walnut Shell Biochar: Characterization, Equilibrium, and Kinetic Studies

**DOI:** 10.3390/molecules30244731

**Published:** 2025-12-10

**Authors:** Izabela Narloch, Grażyna Wejnerowska, Piotr Wojewódzki

**Affiliations:** 1 Department of Food Analysis and Environmental Protection, Faculty of Chemical Technology and Engineering, Bydgoszcz University of Science and Technology, Seminaryjna St. 3, 85-326 Bydgoszcz, Poland; izabela.narloch@pbs.edu.pl; 2 Department of Biogeochemistry, Soil Science and Water Irrigation, Faculty of Agriculture and Biotechnology, Bydgoszcz University of Science and Technology, Kaliskiego Ave. 7, 85-796 Bydgoszcz, Poland; piotr.wojewodzki@pbs.edu.pl

**Keywords:** biochar, biosorption, isotherms, kinetic models, pharmaceuticals, walnut shells

## Abstract

Nonsteroidal anti-inflammatory drugs (NSAIDs), such as salicylic acid (SAL), aspirin (ASP), ketoprofen (KET), and naproxen (NAP), are widely used to relieve pain, fever, and inflammation. For this reason, they are frequently detected in aquatic environments and have a negative impact on many aquatic organisms. In this study, walnut shell biochar (WSB) was used as an adsorbent for the removal of NSAIDs from water. The removal efficiency of pharmaceuticals was highly dependent on various parameters such as pH, contact time, sorbent dosage, and drug concentration. The studies conducted showed that WSB was able to remove as much as about 98% of pharmaceuticals. The maximum adsorption capacities of ASP, SAL, KET, and NAP were 20.92, 33.55, 39.84, and 172.41 mg/g, respectively. The equilibrium data for the investigated drugs showed a better fit to the Freundlich model than the Langmuir model. The pseudo-second-order kinetic model provided the best fit to the kinetic data. The results of the present study show that WSB could be applied as an eco-friendly and cost-effective biosorbent for the removal of drugs from the NSAID group from aqueous solutions.

## 1. Introduction

The nutritional properties of walnuts and the health benefits derived from their consumption contribute to the increase in their production. In addition to the nut kernels being valuable for consumption, their shells can also be used as a potential source of adsorbents. Many scientists have presented promising research results on the use of nutshells for the sorption of emerging pollutants from aqueous solutions, including metals and a wide range of organic compounds. These compounds most commonly include dyes, personal care products, and pharmaceuticals [1,2].

Among pharmaceuticals, nonsteroidal anti-inflammatory drugs (NSAIDs) have been a growing concern in recent years. These compounds have been identified in trace amounts in surface waters and municipal wastewater as well as in wastewater from hospitals and animal farms [3,4,5]. They have negative effects on several aquatic biotas and can also affect human health [6,7]. Therefore, there is increasing interest in removing various pharmaceutical substances from water.

Removal of pharmaceuticals from wastewater can be achieved using several methods, including activated carbon filtration, UV irradiation, reverse osmosis, photocatalytic degradation, ozonation, non-thermal plasma, membrane bioreactors, and advanced oxidation processes. However, these methods are expensive due to the high cost of devices and materials as well as high energy consumption. Of these methods, activated carbon adsorption is generally considered the most effective. However, its industrial application is limited due to its costly production and regeneration processes. To reduce production and operating costs, attempts are being made to develop cheap adsorbents that can be obtained directly from industrial and agricultural waste. Adsorption using alternative, eco-friendly biochar is a promising approach due to its low operating costs, high removal effectiveness, high efficiency, short sorption time, high availability of waste, and potential for reuse. There are many methods available in the literature that use biochar for the sorption of pharmaceuticals such as ketoprofen and diclofenac by the green microalgae *Chlorella* sp. [6]; diclofenac, naproxen, and ketoprofen via chitosan-based magnetic nanosorbents [8]; sulfapyridine, docusate, and erythromycin using biochar derived from cotton gin waste and guayule bagasse [9]; and ciprofloxacin and acetaminophen via banana peel biochar [10]. In turn, the large amount of waste generated from walnut production has fueled interest in this bio-waste as a potential sorbent for removing environmental pollutants. Biochar produced from walnut shells has been used to remove pharmaceuticals, including salicylic acid [11]; naproxen [12]; metformin [13]; amoxicillin, ciprofloxacin, and tetracycline [14]; paracetamol [15]; acetaminophen, sulfapyridine, ibuprofen, and docusate [16], from aqueous solutions. In addition, this biochar has been subjected to various chemical and physical modifications, e.g., activation with phosphoric acid (V) to remove ibuprofen [17] and ciprofloxacin, diclofenac, and sulfamethoxazole [18]; combination with iron (III) nitrate for the removal of diclofenac [19]; modification with iron oxide for the removal salicylic acid, naproxen, and ketoprofen [20]; and modification with ammonia to remove ciprofloxacin [21]. Biochar is a solid by-product produced from biomass [22]. It is produced via biomass thermochemical conversion, which includes pyrolysis, torrefaction, gasification, and hydrothermal processing [23]. Biochar is a multiuse material applied in agriculture, remediation, carbon management, and other industries. The advantageous properties of biochar include improved soil water and nutrient retention, pollutant sorption, and long-term carbon storage. Biochar is generally characterized by a high specific surface area, content of surface functional groups, pH, and porosity [24]. The physicochemical properties (pH, specific surface area, pore size, CEC, volatile matter, and ash and carbon content) of biochar change with pyrolysis temperature and feedstock material [25,26]. The diverse parameters and properties of biochar allow a wide range of applications in various sectors of the economy. Some researchers have proposed methods for improving the adsorption capacity of biochar through chemical modification using, e.g., H_2_SO_4_, HNO_3_, and NaOH [27]. However, these additional steps in biochar production increase costs and preparation time, generate waste, and pose a risk to the operator.

Due to the wide availability of walnut shells and the low cost of obtaining and producing biochar from this waste, in this work, we decided to use this sorbent without chemical or physical modification to study its adsorption potential with respect to four pharmaceuticals, namely, salicylic acid (SAL), aspirin (ASP), ketoprofen (KET), and naproxen (NAP), an endeavor that has not been presented in the literature reports so far. These drugs were selected because they are widely used and considered to be compounds posing a high environmental risk. Several kinetic and isotherm models were applied to describe the adsorption mechanisms. The possibility of biochar regeneration was also investigated, and the economic aspects of this process were presented.

## 2. Results and Discussion

### 2.1. Characterization of Biosorbent

**Figure 1:**
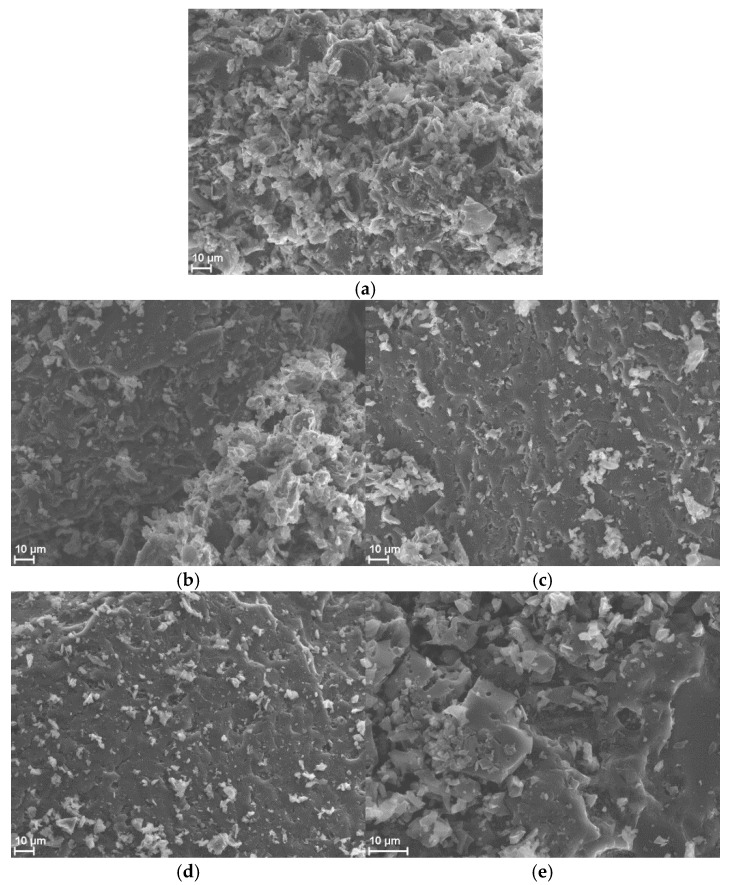
SEM micrographs of WSB (**a**) before adsorption of NSAIDs, (**b**) after biosorption of SAL, (**c**) after biosorption of ASP, (**d**) after biosorption of KET, and (**e**) after biosorption of NAP from aqueous solution.

The morphology of the WSB surface was analyzed using a scanning electron microscope (SEM) before and after pharmaceutical biosorption. The surface morphology of WSB shown in the SEM image reveals the surface structure of the WSB (Figure 1a) before NSAID sorption, showing that it was rough, uneven, and irregular. Moreover, on the surface of the WSB, there are pores of various shapes and sizes, between which there are empty spaces, indicating WSB is very appropriate for removing pollutants. After biosorption of the pharmaceuticals, the number of adsorption species on the WSB surface (Figure 1b–e) decreased, which proves that they were filled by the adsorbed compounds.

Energy-dispersive spectroscopy (EDS) analysis was used to determine the elemental compositions of the WSB surfaces before and after pharmaceutical sorption. The results of the EDS analysis are presented in Table 1. Carbon (~90%) and oxygen (~8%) are the predominant components on the WSB surface relative to the other elements (i.e., Mg, Al, Si, P, S, Cl, K, Ca, Cu, and Fe) determined in the sample. As shown in Table 1, the percentage weight of carbon and oxygen in WSB increased after the adsorption of the tested NSAIDs, ranging from 89.34% to 91.09% for carbon and from 8.14% to 9.64% for oxygen. This indicates effective adsorption of the ASP, SAL, KET, and NAP, which consist primarily of carbon and oxygen, onto the WSB. A similar trend was observed in studies using sorbents of natural origin for removing other pharmaceuticals from water samples [12,19,20,21,22,23,24,25,26,27,28,29].

**Figure 2:**
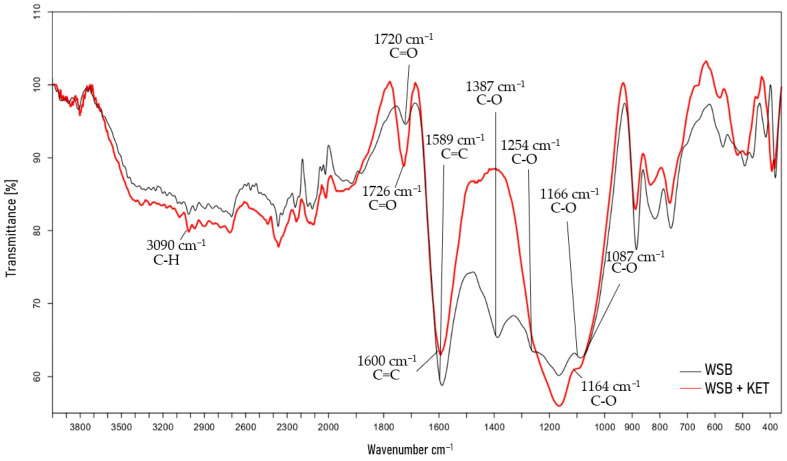
FTIR spectra of WSB before and after KET adsorption.

Fourier transform infrared (FT-IR) spectroscopy was used to identify the main functional groups present in WSB before and after pharmaceutical sorption. The FTIR spectra of the WSB and WSB after sorption (in the example of KET sorption) are shown in Figure 2, while the FT-IR spectra for the remaining pharmaceuticals tested are presented in Figure S1. The FTIR spectra presented some well-defined bands. The FTIR spectrum of the WSB before adsorption shows a sharp peak at 1589 cm^−1^, which may be associated with C=C bonds. In turn, the peaks at 1087, 1166, 1254, and 1387 cm^−1^ were attributed to C-O stretching vibration in the WSB. In the FTIR spectrum of the untreated WSB, the peak at 1720 cm^−1^ is due to C=O stretching, whereas in the FTIR spectrum of the WSB after the KET sorption process, sharpening of the peaks characteristic of the C-H stretching of CH_2_ and CH_3_ groups (3090 cm^−1^); C=O stretching bonds of carboxylic acids, ketones, and aldehydes (1726 cm^−1^); C=C stretching bonds of the aromatic ring (1600 cm^−1^); and C-O stretching bonds (1164 cm^−1^) can be observed, verifying the sorption of the pharmaceutical.

### 2.2. Effect of Adsorption Parameters

#### 2.2.1. Effect of Solution pH

**Figure 3:**
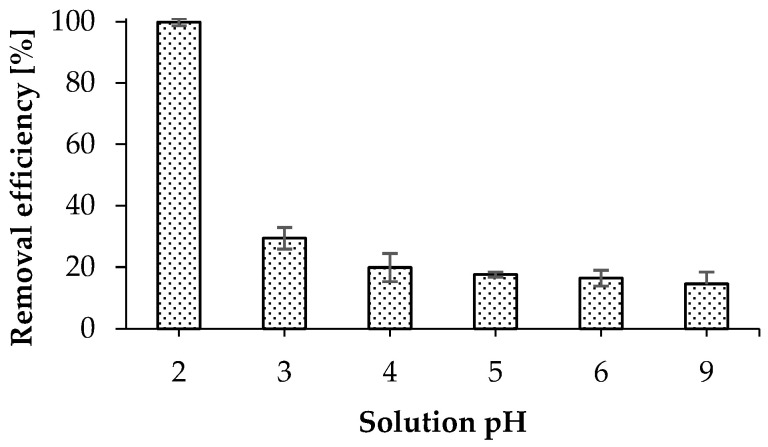
Effect of solution pH on the removal efficiency with respect to KET (conditions: contact time, 1 h; KET concentration, 25 mg/L; temperature, room temperature; sorbent dosage, 1 g/L).

The effect of solution pH on the removal of pharmaceuticals was studied over a pH range of 2–9 using 100 mL of solution and 100 mg of WSB. The initial concentrations of ASP, SAL, KET, and NAP remained 25 mg/L for all compounds. The highest removal efficiencies (96.1–99.8%) of all drugs from aqueous solution were obtained at pH 2. Figure 3 shows the results obtained for KET. A very similar effect of solution pH was observed for the remaining tested compounds (Figure S2). At pH levels higher than 4, the efficiency of removing pharmaceuticals from aqueous solutions decreases to about 20%. A similar tendency regarding the effect of solution pH on the adsorption of the drugs was noted by other authors, including for the adsorption of ketoprofen, sulfamethoxazole, and diclofenac on activated carbon prepared from walnut shell waste [30]; diclofenac, naproxen, ketoprofen, and ibuprofen on active carbon prepared from olive-waste cakes [31]; and salicylic acid on biochar prepared from barley straw [32].

Solution pH has a significant impact on the adsorption process and interactions between sorbent and sorbate surfaces. pH can affect the surface charge of the sorbent as well as the degree of ionization of adsorbate molecules [33]. Hence, a key parameter that can help characterize the sorbent and explain the effect of pH on the adsorption process, especially when considering the influence of electrostatic forces, is the point of zero charge (pH_pzc_). When a solution’s pH is lower than pH_pzc_, the adsorbent’s surface is protonated and positively charged. When the pH of the solution is above pH_pzc_, the adsorbate surface is negatively charged [34].

In these studies, the pH_pzc_ value of the WSB was determined to be 9.0 (Figure S3), which indicates that the WSB surface is positively charged. In turn, taking into account the pK_a_ of the tested pharmaceuticals (Table S1) and the pH of 2, at which the highest sorption efficiency was recorded, it can be stated that NSAIDs occur in cationic or neutral form in solution. According to the literature [35], interactions between adsorbents and adsorbates may include electrostatic interactions, the formation of hydrogen bonds, hydrophobic interactions, complexation, or precipitation. In contrast, the results suggest that the adsorption of the tested pharmaceuticals on biochar occurs mainly as a result of hydrogen bonds and/or hydrophobic interactions, i.e., van der Waals interactions, while there are no electrostatic interactions. Similar results were obtained by Baccar et al. [31], who carried out biosorption of diclofenac, naproxen, ketoprofen, and ibuprofen on activated carbon prepared from olive-waste cakes.

#### 2.2.2. Effect of Biosorbent Dosage

To investigate the effect of biosorbent dosage on the efficiency of removing pharmaceuticals from aqueous solution, various concentrations of the tested compounds were prepared. The differences in concentrations resulted from their different solubilities in water and the possibility of their detection using a spectrophotometer. NAP and KET exhibit low solubility in water, and for them, tests were conducted at concentrations of 30 and 50 mg/L, respectively. SAL and ASP are characterized by higher solubility in water, and in these cases, solutions with a concentration of 200 mg/L were prepared. The sorbent was added in amounts ranging from 50 to 1000 mg to 100 mL of acidified solutions (pH 2). The solutions were stirred for 3 h (500 rpm) at room temperature.

**Figure 4:**
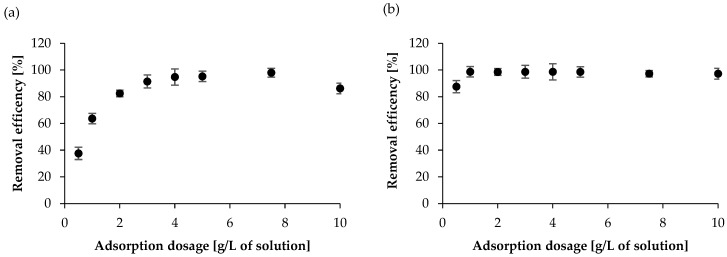
Effect of the dosage of WSB on the removal efficiency for (**a**) SAL at a concentration of 200 mg/L and (**b**) KET at a concentration of 50 mg/L.

As shown in Figure 4 (using the SAL and KET solutions as examples), removal efficiency increases with an increasing WSB dose until a maximum value is reached. In the case of ASP and SAL, the use of 500 mg of WSB was sufficient for nearly complete removal (~95%). However, as expected, in the case of NAP and KET, 100 mg of WSB was sufficient to achieve the maximum degree of adsorption (~98%).

#### 2.2.3. Effect of Initial Concentration

Various concentrations of pharmaceuticals were used to study the effect of initial concentration on the efficiency of their removal from an aqueous solution. The studies were performed in the concentration ranges of 12.5–50 mg/L and 15–30 mg/L for KET and NAP, respectively. However, in the case of the SAL and ASP solutions, concentrations of 50–200 mg/L were prepared. The experiments were performed by adding 100 mg (for KET and NAP) and 500 mg (for ASP and SAL) of the WSB to 100 mL (for KET, ASP, and SAL) and 550 mL (for NAP) solutions for a contact time of 400 min and at a pH of 2.

**Figure 5:**
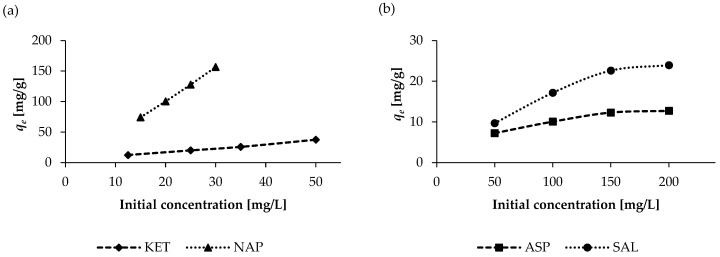
Effect of the initial pharmaceutical concentrations on adsorption capacities (**a**) KET and NAP; (**b**) ASP and SAL.

A graph of the initial drug concentration (mg/L) and equilibrium adsorption capacity q_e_ (mg/g) is presented in Figure 5. As can be seen, increasing the initial drug concentration has a significant impact on adsorption capacity. This is clearly noticeable at low adsorbate concentrations (KET and NAP), where the adsorbent has enough binding sites to accommodate adsorbate molecules. However, at higher drug concentrations (SAL and ASP), saturation of the adsorbent surface can be observed. The 500 mg of WSB could not offer more binding sites for ASP and SAL molecules at their high initial concentrations (150–200 mg/L), resulting in a noticeable decrease in the increase in q_e_ value.

#### 2.2.4. Effect of Contact Time

**Figure 6:**
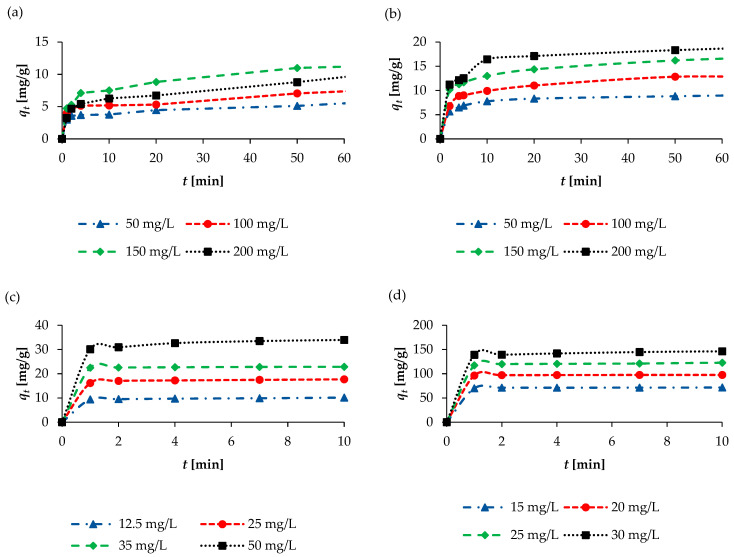
Effect of contact time on adsorption capacity for (**a**) ASP, (**b**) SAL, (**c**) KET, and (**d**) NAP.

The impact of contact time on adsorption capacity with respect to all the pharmaceuticals was examined over a 0–400 min period (Figure S4). The remaining test conditions were the same as those described in Section 2.2.3. As can be seen in Figure 6, in all cases, a very fast adsorption process was observed in the initial step (0–4 min) due to the large number of available active sites on the biosorbent surface. This was followed by a slow increase in the adsorption rate and the establishment of equilibrium.

A more thorough observation shows that in the case of KET, adsorption largely occurs within 1 min. For the lowest KET concentration (12.5 mg/L), adsorption is about 75% (*q_t_* 9.4 mg/g), and it is absorbed the first minute, while after 50 min, adsorption increases to ~89% (*q_t_* ~ 11.1 mg/g). In the case of the highest KET concentration (50 mg/L), about 60% (*q_t_* 30.1 mg/g) is adsorbed within the first minute, and after 50 min of the process, the adsorption degree increases to about ~72% (*q_t_* 36.1 mg/g). Therefore, the 50 min sorption time was considered the equilibrium time. The adsorption of NAP, which was studied at concentrations ranging from 15 to 30 mg/L, proceeded similarly. In the case of NAP, the maximum sorption efficiency was reached as early as the first minute (WSB mass 0.1 g, solution volume 550 mL); this value was approximately 84–87% (*q_t_* 69.8–139 mg/g). Depending on the NAP concentration, the equilibrium time (*t_eq_*) for the lowest concentration (15 mg/L) was 4 min (86%, *q_t_* 71.1 mg/g), while the *t_eq_* for the highest concentration was 50 min (93%, *q_t_* 153.6 mg/g).

The sorption process studies for SAL and ASP were conducted at higher concentrations (50–200 mg/L), and in these cases, a much slower sorption process was observed. After the first minute of adsorption, for the lowest concentration used (50 mg/L), the adsorption degrees were 30% and 42% (*q_t_* 3.0 and 4.2 mg/g) for ASP and SAL, respectively. However, for the highest concentration (200 mg/L), after the first minute of adsorption, the degree was much lower and amounted to 8.1% and 22.5% (*q_t_* 3.2–9.0 mg/g) for ASP and SAL, respectively. The *t_eq_* for ASP and SAL (for concentration 50 mg/L) was 400 min, at which point the removal rates were 72.5% and 97% (*q_t_* 7.25–9.69 mg/g) for ASP and SAL, respectively.

### 2.3. Adsorption Kinetics Studies

**Figure 7:**
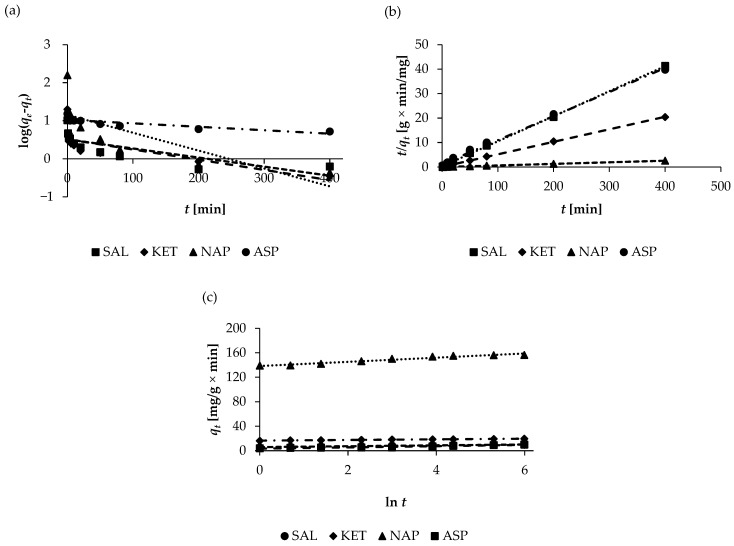
Adsorption kinetics of pharmaceuticals: (**a**) pseudo-first-order kinetic model, (**b**) pseudo-second-order kinetic model, and (**c**) Elovich model.

Adsorption kinetics are an important parameter in describing the rate and mechanism of adsorption processes. The sorption kinetics of the selected pharmaceuticals on WSB were examined using four commonly used kinetic models: pseudo-first-order, pseudo-second-order, Elovich, and intra-particle diffusion models. Kinetic experiments were conducted on solutions containing the analytes’ initial concentrations of 100, 50, 30, and 25 mg/L for ASP, SAL, NAP, and KET, respectively, and over a range of 0 to 400 min. The kinetic parameters of ASP, SAL, NAP, and KET are presented in Table 2. The fitting of the experimental data regarding the pharmaceuticals’ biosorption for the pseudo-first-order, pseudo-second-order, and Elovich models is shown in Figure 7. The correlation coefficients (*R*^2^) and agreement between the experimental (*q_e_*) and calculated data (*q_cal_*) were used as criteria to create a kinetic model of pharmaceutical adsorption. The correlation coefficient for the pseudo-second-order equation (*R*^2^ > 0.9956) is much higher than that obtained for the pseudo-first-order equation (*R*^2^ ≤ 0.5939). Moreover, the experimental equilibrium sorption capacity (*q_e_*) of the pseudo-second-order kinetic model was almost identical to the calculated equilibrium sorption capacity (*q_cal_*). The data obtained (Table 2) show that the sorption of all pharmaceuticals best follows a pseudo-second-order kinetic model. This model assumes that chemisorption is the rate-limiting step in adsorption [36], in contrast to physisorption, which is the basis of the pseudo-first-order model, which limits the diffusion of sorbate into the sorbent and is based on van der Waals forces and electrostatic and hydrophobic interactions [37]. The approximate *k*_2_ rates for most analytes decrease with an increase in concentration (Table S2) owing to the increased number of active zones on the adsorbent’s surface at the beginning of sorption; this is followed by a decrease in the rate due to the high competition of molecules (at high analyte concentrations) occupying the available active zones on the adsorbent’s surface [38]. The pseudo-second-order kinetic model is in agreement with other methods of sorption using biosorbents in studies regarding pharmaceuticals [31,39,40]. In the case of the Elovich kinetic model, high values of the fitting coefficient (*R*^2^ > 0.9202) were also obtained, indicating that the biosorption of the pharmaceuticals on the WSB involves chemisorption, which consists of the formation of an ionic or covalent chemical bond between the adsorbent and the adsorbate. For most pharmaceuticals, the Elovich desorption rate (*β*) decreases with an increasing initial concentration, a phenomenon related to the degree to which the surface of the sorbent is covered by adsorbate molecules during chemisorption. Similarly, the Elovich sorption rate (*α*) decreases with an increasing initial analyte concentration, indicating multilayer adsorption (Table S2). In addition, the high value of α suggests that biosorption occurs rapidly in the initial minutes. However, the experimental data for all three kinetic models best correlate with the pseudo-second-order model.

**Figure 8:**
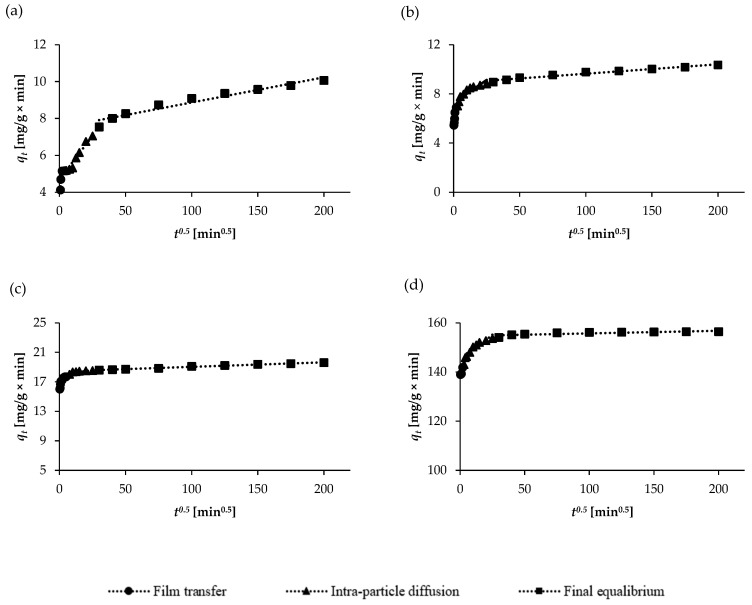
The intra-particle diffusion of (**a**) ASP, (**b**) SAL, (**c**) KET, and (**d**) NAP on WSB.

In order to effectively investigate the diffusion mechanisms of the adsorption of the pharmaceuticals on the adsorbent, the intra-particle diffusion model was used. The parameters of the intra-particle diffusion models for all the analytes are summarized in Table 3. Wide deviation of the constant associated with the thickness of the boundary layer (*C*) values starting from zero (5.452–8.926 for ASP, 4.092–4.604 for KET, 137.760–155.061 for NAP, and 3.738–7.695 for SAL) and non-linear plots (which do not pass through origins) over time suggested the presence of intra-particle diffusion and other adsorption mechanisms. Figure 8 illustrates the intra-particle diffusion of the selected pharmaceuticals, which occurred in three stages: film diffusion, intra-particle diffusion, and mass action. The first stage corresponds to the diffusion of the adsorbate in the boundary layer (surface film). This step is very short (about 5 min), occurs rapidly, and is externally diffusion-controlled. In turn, in the second stage, the pharmaceutical molecules penetrate the interior of the particles of the sorbent through intra-particle diffusion, wherein saturation occurs. Finally, in the third stage, the adsorption of the analytes occurs on the active regions of the internal and external pore surfaces, during which the equilibrium state is established. The equilibrium state is reached when the intra-particle diffusion stage slows down due to the very low residual adsorbate concentration, which results in a change in the number of active sites on the sorbent surface. The presence of a three-step linear region suggests that the intra-particle diffusion model is not the only rate-limiting step. Similarly, other authors have reported that three-stage kinetics have been observed for the adsorption of naproxen onto walnut shell biochar [12], ketoprofen and aspirin adsorption using alga-derived biochar [41], and the adsorption of salicylic acid onto banana peel biochar [42].

### 2.4. Adsorption Isotherm Studies

The Langmuir and Freundlich isotherm models were used to fit the adsorption data to understand the adsorption mechanisms. In general, the Langmuir model is based on the assumption that there is monolayer coverage and indicates a homogeneous surface, whereas the Freundlich equation suggests that the adsorbent consists of heterogeneous surfaces with different binding sites [43]. Linear regression is commonly used to determine the best-fitting isotherm, and the applicability of isotherm equations is compared by evaluating the correlation coefficient, R^2^. The experiments were performed under optimum conditions while varying the concentrations of SAL, ASP, KET, and NAP. The overall results for the Langmuir and the Freundlich isotherm models are shown in Table 4.

The Langmuir adsorption isotherm is the most widely used model for describing the equilibrium between an adsorbate and an adsorbent. This isotherm describes the ideal situation in which the adsorbates are attached onto a homogenous surface on the adsorbent in a monolayer without interaction between the adsorbate molecules. The adsorption energy of each adsorbate is identical to that of the other absorbates and independent of the adsorbent surface. The linearity of the Langmuir isotherm was determined according to Equation (6).

**Figure 9:**
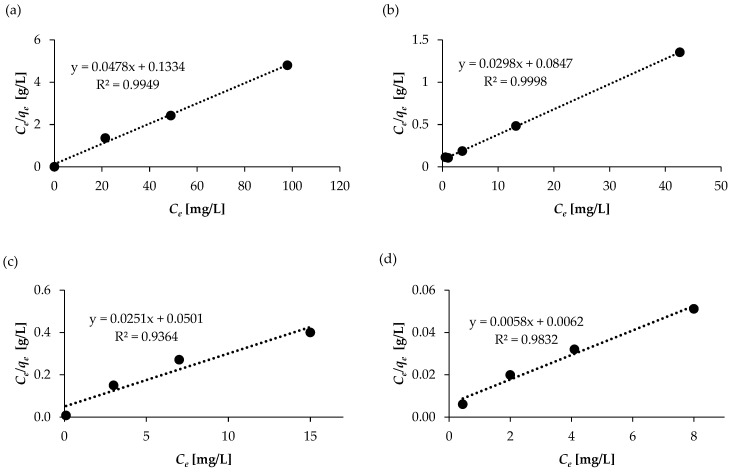
Langmuir isotherm models for the adsorption of NSAIDs on WSB: (**a**) ASP, (**b**) SAL, (**c**) KET, and (**d**) NAP.

The values of Langmuir constants *q*_max_ and *K*_L_ were obtained from the intercept and the slope of the linear plot *C*_e_/*q*_e_ against *C*_e_ (Figure 9 and Table 4). The plot of the Langmuir equation showed a good correlation (*R*^2^ > 0.99), indicating monolayer-type adsorption of the SAL and ASP. In turn, the values of *R*^2^ obtained for KET and NAP were slightly lower, amounting to 0.9364 and 0.9832, respectively. This may indicate that the process may occur differently.

The highest maximum adsorption capacity (*q_max_*) value, 172.41 mg/g, was obtained for NAP. The highest *K_L_* value, 0.936, was also obtained for this component, indicating strong interactions between the sorbent surface and NAP. This is probably related to the high hydrophobicity of NAP and the presence of methoxy and carboxyl functional groups and two aromatic rings. For the remaining compounds, *q_max_* ranged from 20.92 to 39.84 mg/g, and *K_L_* values were also lower, ranging from 0.352 to 0.501. This may be due to their less favorable structures (Table S1), which lead to increasingly weaker π–π interactions and hydrogen bonds between the tested compounds and the WSB surface. The results indicate that there is a difference in the adsorption affinity of the WSB toward the pharmaceuticals tested.

Based on the Langmuir model equation, the dimensionless separation factor *R_L_* (equilibrium parameter, Equation (7)) was determined. The estimate of *R_L_* suggests the Langmuir isotherm that is favorable for adsorption (0 < *R_L_* <1), results in irreversible adsorption (*R_L_* = 0), is linear (*R_L_* = 1), or is unfavorable for adsorption (*R_L_* > 1). For all the tested compounds, the values of *R_L_* were <1. However, the closer the *R_L_* value is to 0, the more favorable the adsorption process. Among the tested compounds, NAP is characterized by the lowest *R_L_* value: 0.01. Ultimately, the *R_L_* values confirmed that all drugs were effectively absorbed by WSB and the process was spontaneous.

**Figure 10:**
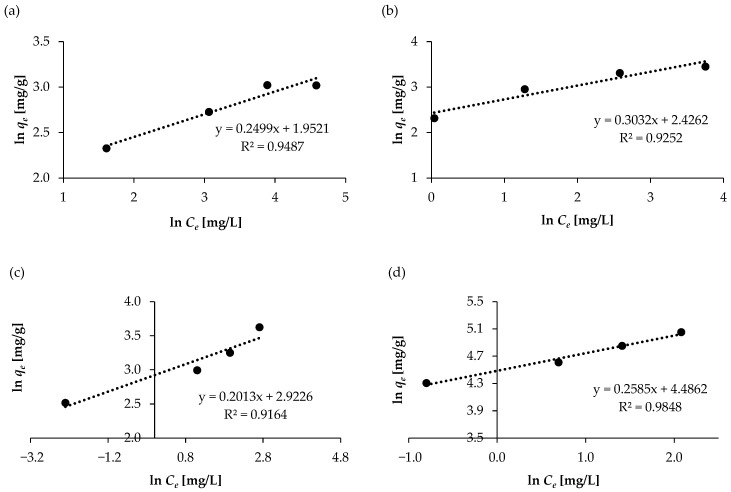
Freundlich isotherm models for the adsorption of NSAIDs on WSB: (**a**) ASP, (**b**) SAL, (**c**) KET, and (**d**) NAP.

The Freundlich isotherm differs from the Langmuir isotherm in that it describes the heterogeneous adsorption of molecules at different positions and adsorption energies. In the case of the Freundlich isotherm models, *K_F_* and *n* constants were determined from the intercept and slope of the plot between ln *q_e_* and ln *C_e_*. Table 4 presents the values of the adsorption constants, and Figure 10 presents isotherms for the Freundlich model. The *K_F_* constant indicates adsorption capacity, while n indicates adsorption effectiveness. The *K_F_* values were 11.087, 7.044, and 18.272 for SAL, ASP, and KET, respectively. However, a much higher value of the *K_F_* constant, 88.783, was obtained for NAP. For all four compounds, *n* is >1 (favorable adsorption is when 1 < *n* < 10), indicating that the sorption process is favorable and adsorption occurs in the physical system [44].

For all the tested pharmaceutical products, *R*^2^ > 0.9 (0.9164–0.9848) was obtained. These values are lower than those obtained with the Langmuir model. In most cases (except NAP), the Langmuir equation is the best-fitting model for the studied systems, indicating adsorption of SAL, ASP, and KET in the monolayer. This implies that the adsorption sites on the solid’s surface are all energetically equivalent and that each site can fix only one molecule.

### 2.5. Comparison with Other Adsorbents

The efficiency of SAL, ASP, KET, and NAP removal by means of WSB was compared with that for other biosorbents previously reported in the literature (Table 5). For this purpose, q_max_ determined from isotherm model fitting at room temperature was used. In the case of the above-mentioned pharmaceuticals, in this study, the *q_max_* was 33.55, 20.92, 39.84, and 172.41 mg/g for SAL, ASP, KET, and NAP, respectively. The differences in absorption capacity of the selected biosorbents described in the literature may be a result of various factors, such as the type of biosorbent, its parameters and modifications, the physico-chemical properties of the compounds, and the conditions of the sorption process (e.g., pH, temperature, contact time, and concentration of compound).

### 2.6. Desorption and Regeneration Studies

The reusability of a adsorbent has a significant impact on its economic and ecological effectiveness. The efficiency of the desorption of NSAIDs from WSB was tested by using desorption agents such as MeOH, ACN, and water. Examples of the results regarding the desorption efficiency of KET with respect to WSB are presented in Table 6. Based on the results, it can be concluded that MeOH and ACN are very effective desorption agents for these compounds. The other tested agents, such as distilled water with different pH levels (2, 4, 8, and 10) at room temperature and hot distilled water (pH 6.5; 80 °C), did not show satisfactory desorption efficiencies. Based on the results obtained, MeOH was selected for further testing.

**Figure 11:**
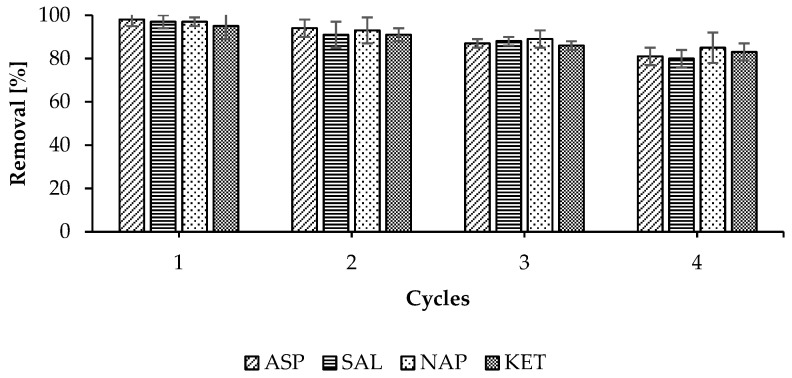
NSAID regeneration study regarding WSB.

The reusability of the WSB was determined using the four adsorption–desorption cycles. NSAIDs were desorbed from the biosorbent surface by putting WSB into MeOH. As shown in Figure 11, MeOH desorbed ASP, SAL, NAP, and KET from the WSB with a removal performance staying between 80% and 98%. The noticeable slight decrease (approximately 20%) in the efficiency of the removal of the drug from the WSB surface in the fourth cycle was probably caused by partial removal of the finest carbon fraction during its filtering between individual cycles. The presented results prove that it is possible to use this sorbent multiple times, which proves that WSB has great potential to be a cost-effective sorbent. However, using methanol, a toxic solvent, for biochar regeneration increases the costs associated with its purchase and regeneration/disposal (especially on an industrial scale). Therefore, choosing the appropriate method for regenerating used biochar is a significant economic aspect of the entire pharmaceutical disposal process.

### 2.7. Estimation of the Cost of WSB Preparation

Analysis of the cost of developing effective biosorbents for the removal of contaminants is an essential part of the implementation of water treatment technologies. Sorbent production accounts for the largest share of the total cost of the remediation process. The cost of sorbent production is mainly related to collection, crushing, washing, carbonization, and other overhead factors such as activation and neutralization when these steps are used in sorbent production. All of the above-mentioned criteria play a part in influencing the cost of the biosorbent, while our proposal for obtaining a biosorbent only takes into account collection, crushing, and carbonization, and it is presented in Table 7. To produce 1 kg of WSB, 3.85 kg of walnut shells is needed. The total cost of producing 1 kg of WSB in laboratory conditions was USD 2.97, while on an industrial scale, the cost of producing this biochar might be much lower; it may be comparable to the cost of obtaining 1 kg of commercially available activated carbon (USD 1.5–3—the price depends on the raw material).

## 3. Materials and Methods

### 3.1. Chemicals

Aspirin (ASP), salicylic acid (SAL), ketoprofen (KET), and naproxen (NAP) were obtained as high-purity certified reference materials from Sigma Aldrich (St. Louis, MO, USA). The physico-chemical properties of ASP, SAL, KET, and NAP are shown in Table S1. Methanol (MeOH) and acetonitrile (ACN) were HPLC-grade (Honeywell International Inc., Charlotte, NC, USA). The sodium chloride (NaCl), sodium hydroxide (NaOH), and hydrochloric acid (HCl) obtained from Chempur (Piekary Śląskie, Poland) were analytical-reagent-grade.

### 3.2. Preparation of Biosorbent

Walnut shells were obtained from a local farm (Bydgoszcz, Poland). Prior to processing, the shells were crushed into smaller particles using a hand mortar (Figure S5). The walnut shell biochar was produced via pyrolysis (600 °C) of air-dried feedstock biomass (moisture 4.9%) under atmospheric pressure in a muffle furnace FCF 22 M (Czylok, Jastrzębie-Zdrój, Poland). Process execution time was 60 min. The obtained WSB was ground and homogenized in a ball mill PM100 (RETSCH GmbH, Haan, Germany). Parameters of milling process were as follows: a time of 10 min, at 400 rpm, with ten zirconium balls in the milling chamber. The mean size distribution of ground WSB particles was as follows: 2.0–0.05 mm—64.49%; 0.05–0.002 mm—32.23%; and <0.002 mm—3.28%. The average yield of WSB was 26.42% ± 0.38, and the bulk density of ground WSB was 0.575 g/cm^3^ ± 0.019. The prepared biochar was stored in a closed glass bottle.

### 3.3. Characterization of Biosorbent

#### 3.3.1. pH at Point of Zero Charge (pH_pzc_)

pH_pzc_ was determined according to the method reported by Maaloul et al. [55]. Specifically, 20 mL of 0.05 M NaCl solution was poured into flasks, and the pH of NaCl solutions was adjusted from 2 to 10 by adding 0.01 M HCl or 0.01 M NaOH solutions. Then, 50 mg of the biochar was immersed in the solutions and stirred (200 rpm) for 24 h at room temperature, and then the pH measurement was conducted. The pH_pzc_ was obtained from a plot of (∆pH = pH_initial_ − pH_final_) vs. pH_initial_, and its value corresponds to the intercept point of the curve from the initial pH axis.

#### 3.3.2. Fourier-Transform Infrared Spectroscopy (FT-IR)

The structural characterization of the biochar was performed by using a Bruker ALPHA Fourier-transform infrared spectrophotometer (FT-IR) (Bruker, Berlin, Germany), using an attenuated total reflection technique (4000–360 cm^−1^ wavelength range).

#### 3.3.3. Scanning Electron Microscopy–Energy-Dispersive Spectroscopy (SEM-EDS)

The surface and morphological properties of the biochars (raw, after adsorption, and after desorption) were characterized using a scanning electron microscope (SEM/FIB CrossBeam 540, Zeiss, Oberkochen, Germany) equipped with an energy-dispersive X-ray microanalysis (EDS) probe (Oxford Instruments, Oxford, UK).

#### 3.3.4. Biosorption Experiments

- Batch Adsorption Experiments

Adsorption efficiency of ASP, SAL, KET, and NAP on WSB was evaluated using a batch system at room temperature. To determine the biosorption behavior of tested pharmaceuticals, several key parameters were determined to optimize the sorption process. These detailed studies included pH solutions, biosorbent dosage, initial drug concentrations, and contact time. During the sorption experiments, a small quantity of the solution was filtered using 0.2 µm nylon syringe filters and analyzed with a JENWAY 7315 UV-Vis spectrophotometer (Bibby Scientific Ltd., Stone, Staffs, UK). The absorbance data at wavelengths of 294, 276, 262, and 264 nm for SAL, ASP, KET, and NAP, respectively, were used to calculate the quantity of pharmaceuticals in the solution. All experiments were conducted in triplicate.

The uptake of pharmaceuticals (mg/g) was calculated based on the following equation (Equation (1)):

(1) $$q_{e}=\frac{\left(C_{0}-C_{t}\right)V}{m}$$

where C_0_ and C_t_ (mg/L) are the concentrations of the adsorbate in aqueous solution at the initial and final points (after contact time), V (L) is the volume of the solution, and m (g) is the mass of the biosorbent.

- Adsorption Kinetics

In order to investigate the mechanism of the sorption process, pseudo-first-order, pseudo-second-order, Elovich, and intraparticle diffusion models were applied to test the adsorption rate data.

The pseudo-first-order rate model is expressed in Equation (2), as per the equation developed by Lagergren [56]:

(2) $$\ln\left(q_{e}-q_{t}\right)=lnq_{e}-k_{1}\;\times t$$

where $q_{e}\;and\;q_{t}$ are the quantity of sorbed analytes in the qualibrium state and at time t (mg/g), t is time, and $k_{1}$ is the pseudo-first-order rate constant (1/min).

The pseudo-second-order rate model is given by Equation (3) [57]:

(3) $$\frac{t}{q_{t}}=\frac{1}{k_{2}\;\times q_{e}^{2}}+\frac{t}{q_{e}}\;$$

where $k_{2}$ is the rate constant of the pseudo-second-order (g/mg × min).

The Elovich model is expressed as follows (Equation (4)) [58]:

(4) $$q_{t}=\frac{1}{\beta }\ln\left(\alpha \;\times \;\beta \right)+\frac{1}{\beta }\ln\left(t\right)$$

where α is the initial sorption rate (mg/g × min), and β is an indicator of desorption (g/mg).

The intraparticle diffusion model is expressed as follows (Equation (5)) [59]:

(5) $$q_{t}=k_{int}t^{0.5}+C$$

where $k_{int}$ is the intraparticle diffusion rate constant (mg/g × min), and *C* is a constant associated with the thickness of the boundary layer (mg/g).

- Adsorption Isotherms

To investigate the adsorption process [60], the experimental results were interpolated using the Lanqumir model. The following equation (Equation (6)) depicts the linear version of the Langmuir isotherm model:

(6) $$\frac{C_{e}}{q_{e}}=\frac{C_{e}}{q_{max}}+\;\frac{1}{K_{L}\;\times q_{max}}\;$$

where *C*_e_ (mg/L) represents the equilibrium concentration of the pharmaceutical in the solution, *q*_e_ (mg/g) is the adsorption capacity of WSB at equilibrium, and *q*_max_ (mg/g) and *K_L_* (L/mg) represent the monolayer (maximum) adsorption capacity and energy of adsorption, respectively.

The dimensionless constant separation factors, *R*_L_, was determined using the following Equation (7):

(7) $$R_{L}=\frac{1}{\left(1+K_{L}\times C_{0}\right)}$$

where *C*_0_ is the initial pharmaceutical concentration (mg/L), and *K*_L_ is the Langmuir constant (L/mg).

The following equation (Equation (8)) was used to obtain the linear form of the Freundlich isotherm:

(8) $$\ln q_{e\;}=lnK_{F}+\;\frac{1\;}{n}\ln C_{e}\;$$

where *K*_F_ is the Freundlich constant ((mg/g) × (L/mg)^1/*n*^), *n* is the degree of nonlinearity and gives an estimate of adsorption strength, *C*_e_ is the adsorbate concentration at equilibrium (mg/L), and *q*_e_ is the number of pharmaceuticals adsorbed on the WSB (mg/g).

### 3.4. Desorption Studies

To examine the reusability of the developed biosorbent, the WSBs after drug adsorption were subjected to desorption experiments with different desorption agents, including MeOH, ACN, distilled water with various pH levels (2, 4, 8, and 10), and hot distilled water (pH 6.5, 80 °C). For this purpose, the 0.5 g WSB was immersed in 5 mL of each desorption agent, and the mixture was kept in a shaker (500 rpm) for 1 h. After filtration, the adsorbate solution was quantified by using UV-Vis spectrophotometry. The desorbate WSB was used in the next cycle of the adsorption–desorption study.

## 4. Conclusions

The biosorption of pharmaceuticals such as salicylic acid, aspirin, ketoprofen, and naproxen onto walnut shell biochar was studied. In this work, structural characterization of the sorbent, kinetics, adsorption, and regeneration were investigated. Kinetics pertaining to the removal of NSAIDs were obtained and fitted to different kinetics models. Kinetics data were best fitted by the pseudo-second-order model, and the results indicated that the walnut shell biochar adsorbent exhibited excellent performance in the removal of used drugs from water. The equilibrium data for all the pharmaceuticals best fitted the Langmuir model, with a correlation coefficient for all compounds > 0.9364. This suggests a monolayer coating of the sorbent surface by the NSAIDs. The research results prove that walnut shell biochar is a low-cost biosorbent characterized by high sorption capacities (*q_max_* was 33.55, 20.92, 39.84, and 172.41 mg/g for SAL, ASP, KET, and NAP, respectively). Thus, WSB could be a more efficient and economic adsorbent with greater application potential toward NSAID molecules relative to biochar produced from other types of waste.

## Figures and Tables

**Table 1:** Elemental composition of WSB before and after adsorption of the pharmaceuticals.

WSB		WSB + ASP		WSB + SAL		WSB + NAP		WSB + KET	
Element	Weight %	Element	Weight %	Element	Weight %	Element	Weight %	Element	Weight %
C	89.34	C	89.94	C	90.34	C	91.09	C	89.53
O	8.14	O	8.87	O	8.85	O	8.28	O	9.64
Mg	0.07	Mg	0.06	Mg	0.05	Mg	0.04	Mg	0.06
Al	0.01	P	0.12	Si	0.03	Si	0.03	Si	0.04
Si	0.01	S	0.04	P	0.06	P	0.07	P	0.07
P	0.10	Cl	0.33	S	0.03	S	0.03	S	0.03
S	0.03	K	0.15	Cl	0.09	Cl	0.15	Cl	0.24
Cl	0.05	Ca	0.39	K	0.14	K	0.07	K	0.05
K	1.80	Fe	0.03	Ca	0.30	Ca	0.18	Ca	0.25
Ca	0.36	Cu	0.09	Cu	0.10	Fe	0.01	Cu	0.08
Cu	0.09					Cu	0.06		
Total:	100.00	Total:	100.00	Total:	100.00	Total:	100.00	Total:	100.00

**Table 2:** Biosorption kinetic parameters for ASP, SAL, NAP, and KET on WSB.

Analyte	*C*_0_ [mg/L]	*q_e_* [mg/g]	Pseudo-First-Order			Pseudo-Second-Order			Elovich		
			*q_cal_* [mg/g]	*k*_1_ [1/min]	*R* ^2^	*q_cal_* [mg/g]	*k*_2_ [g/mg × min]	*R* ^2^	*α* [mg/g × min]	*β* [g/mg]	*R* ^2^
**ASP**	100	15.28 ± 0.98	10.58 ± 0.32	0.0021 ± 0.0004	0.7318	14.16 ± 0.35	0.0086 ± 0.0008	0.9956	33.6 ± 3.5	0.9985 ± 0.0116	0.9202
**SAL**	50	10.31 ± 0.82	3.23 ± 0.22	0.0055 ± 0.0009	0.5939	9.77 ± 0.17	0.0405 ± 0.0018	0.9998	3683.8 ± 241.3	1.4349 ± 0.0265	0.9729
**KET**	25	19.96 ± 0.37	3.27 ± 0.05	0.0062 ± 0.0005	0.5726	19.57 ± 0.55	0.0317 ± 0.002	0.9998	2.9 × 10^13^ ± 2.1 × 10^12^	1.9209 ± 0.1504	0.9726
**NAP**	30	156.44 ± 2.32	14.27 ± 0.36	0.0108 ± 0.00147	0.6390	156.25 ± 3.25	0.0114 ± 0.0011	1.0000	1.7 × 10^18^ ± 2.0 × 10^17^	0.2942 ± 0.02	0.9545

**Table 3:** Intra-particle diffusion model parameters for adsorption of pharmaceuticals.

Analyte	*k*_p1_ [mg/g/min^0.5^]	*k*_p2_ [mg/g/min^0.5^]	*k*_p3_ [mg/g/min^0.5^]	*C*_1_ [mg/g]	*C*_2_ [mg/g]	*C*_3_ [mg/g]	R_1_^2^	R_2_^2^	R_3_^2^
**ASP**	0.7632	0.0480	0.0074	5.452	7.666	8.926	0.8613	0.9095	0.9705
**SAL**	0.0669	0.0046	0.0008	4.092	4.480	4.604	0.9678	1.0000	0.9932
**KET**	0.2704	0.0128	0.0058	16.463	18.212	18.466	0.7117	1.0000	0.9871
**NAP**	2.0013	0.3313	0.0075	137.760	145.624	155.060	0.9382	0.8997	0.7894

**Table 4:** Adsorption isotherm parameters for the removal of NSAIDs by WSB.

Model	Adsorption Parameters	ASP	SAL	KET	NAP
Langmuir	*K*_L_ (L/mg)	0.358 ± 0.027	0.352 ± 0.018	0.501 ± 0.045	0.936 ± 0.095
	*q*_max_ (mg/g)	20.92 ± 0.324	33.55 ± 0.361	39.84 ± 0.954	172.41 ± 4.583
	*R* ^2^	0.9949	0.9998	0.9364	0.9832
	*R*_L_	0.053 ± 0.001	0.102 ± 0.006	0.138 ± 0.014	0.010 ± 0.002
Freundlich	*K*_F_ (mg/g)(L/mg)^1/*n*^	7.044 ± 0.157	11.087 ± 0.284	18.272 ± 0.833	88.783 ± 9.985
	*n*	4.002 ± 0.098	3.220 ± 0.040	5.308 ± 0.249	3.868 ± 0.265
	*R* ^2^	0.9487	0.9252	0.9164	0.9848

**Table 5:** Comparison of adsorption performance of several biosorbents reported in the literature for the removal of ASP, SAL, KET, and NAP.

Biosorbent	NSAIDs	*q*_max_ [mg/g]	Conditions	Reference
Chitosan-modified waste tire rubber	NAP	2.3	pH: 6 t_eq_: 60–120 min trt: -	[45]
Peanut shells	NAP	55.1	pH: 7 t_eq_: 120 min trt: chemical modification (H_2_O_2_)	[34]
Orange peel	NAP	28.1	pH: 4 t_eq_: 30 min trt: -	[46]
Bamboo biochar	NAP	56.5	pH: 3 t_eq_: 90 min trt: -	[47]
Walnut shell biochar	NAP	58.8	pH: 6.84 t_eq_: 240 min trt: -	[12]
Olive-waste cake biochar	NAP KET	39.5 24.7	pH: 2.01 t_eq_: 26 h trt: chemical modification (H_3_PO_4_)	[31]
Rice husk	ASP	47.0	pH: 2 t_eq_: 180 min treatment: -	[48]
Coffee waste biochar	ASP	490.1	pH: - t_eq_: 30–60 min trt: chemical modification (H_3_PO_4_)	[49]
Sugarcane bagasse biochar	ASP	32.7	pH: 4 t_eq_: 120 min trt: -	[50]
Green microalgae, *Chlorella* sp.	KET	0.6	pH: 6 t_eq_: 60 min trt: -	[6]
Zeolites	KET	1.8	pH: 5 t_eq_: 20–30 min trt: chemical modification (Cetylpyridinium chloride or Arquad^®^ 2HT-75)	[51]
Babassu coconut husk biochar	KET	89.2	pH: 2 t_eq_: 300 min trt: -	[52]
Babassu coconut husk biochar	KET	79.1	pH: 2 t_eq_: 300 min trt: physical modification (sonication)	[52]
*Campomanesia* *guazumifolia* bark	KET	158.3	pH: 2 t_eq_: 180 min trt: chemical modification (H_2_SO_4_)	[53]
Banana peel	SAL	9.8	pH: 3.3 t_eq_: 14 h trt: -	[54]
Barley straw biochar	SAL	189.2	pH: 3 t_eq_: 11 h trt: -	[32]
Walnut shell biochar	ASP	33.6	pH: 2	This work
	SAL	20.9	t_eq_: 4 min for KET, NAP	
	KET	39.8	t_eq_: 400 min for SAL, ASP	
	NAP	172.4	trt: -	

t_eq_—equilibrium time; trt—treatment.

**Table 6:** Efficiency of desorption (%) of KET from the WSB (conditions: 100 mL solution (C_0_ = 25 mg/L), 1 h, 0.5 g WSB, and 5 mL of desorption agent).

Desorption Agent	Desorption Efficiency (%)
MeOH	98 ± 2
ACN	65 ± 6
H_2_O, pH 2	7 ± 2
H_2_O, pH 4	11 ± 4
H_2_O, pH 8	14 ± 3
H_2_O, pH 10	15 ± 1
H_2_O, pH 6.5, 80 °C	17 ± 3

**Table 7:** Cost estimation of 1 kg WSB production.

Particulars	Subsections	Cost Breakdown	Total Cost ($)
Raw-material processing	Collection of raw material	Collected from local farm	0
	Size reduction cost	Size reduction was performed manually	0
Preparation of WSB	Carbonization cost	[Hours × unit × unit cost] 1.75 h × 4.5 kW × 0.29 $	2.28
	Size reduction cost	[Hours × unit × unit cost] × 7 * 0.33 h × 1.25 kW × 0.29 $ × 7	0.42
Net cost			2.70
10% of overall cost			0.27
Total cost			2.97

* Number of batches for the grinder.

## Data Availability

The original contributions presented in this study are included in the article and Supplementary Materials. Further inquiries can be directed to the corresponding author.

## Supplementary Materials

The following supporting information can be downloaded at https://www.mdpi.com/article/10.3390/molecules30244731/s1. Figure S1. FTIR spectra of WSB before and after (a) NAP, (b) SAL, and (c) ASP adsorption. Figure S2. Effect of the solution pH on the removal efficiency of (a) SAL, (b) ASP, and (c) NAP (conditions: contact time 1 h, drug concentration 25 mg/L, room temperature, sorbent dosage 1 g/L). Figure S3. Plot of pH point of zero charge of WSB. Figure S4. Effect of the contact time on adsorption capacity of (a) ASP, (b) SAL, (c) KET, and (d) NAP. Figure S5. The raw, crushed walnut shells (left) and obtained WSB prior grounding (right). Table S1. Characterization of tested pharmaceuticals. Table S2. Biosorption kinetic parameters for adsorption of ASP, SAL, NAP, and KET onto WSB.
