# Supplementary material for: Biosorption of Aspirin, Salicylic Acid, Ketoprofen, and Naproxen in Aqueous Solution by Walnut Shell Biochar: Characterization, Equilibrium, and Kinetic Studies
Source: Molecules. 2025 Dec 10;30(24):4731. doi: 10.3390/molecules30244731 (PMC12735971; doi:10.3390/molecules30244731)
Supplement: Supplementary file 1 [file molecules-30-04731-s001.zip › molecules-4001483-supplementary.pdf]

Supplementary Materials

Biosorption of Aspirin, Salicylic Acid, Ketoprofen, and Naproxen in Aqueous Solution by Walnut Shell Biochar: Characterization, Equilibrium, and Kinetic Studies

Izabela Narloch, Grażyna Wejnerowska and Piotr Wojewódzki

a)

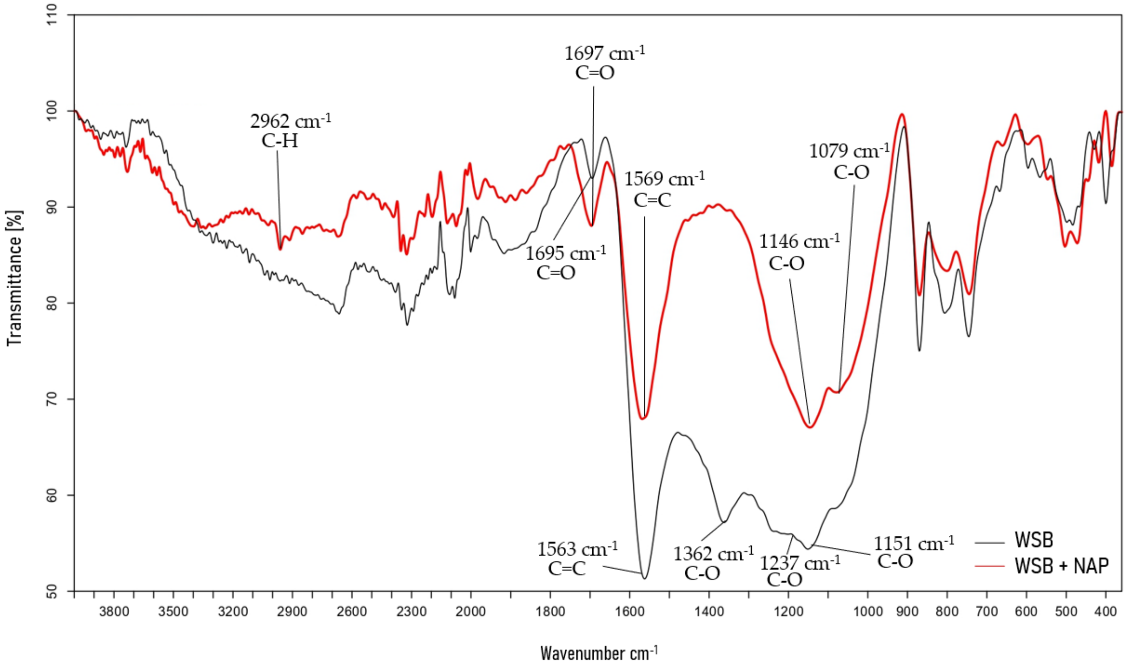

Transmittance [%]
Wavenumber cm-1
110
100
90
80
70
60
50
3800
3500
3200
2900
2600
2300
2000
1800
1600
1400
1200
1000
900
800
700
600
500
400
2962 cm-1
C-H
1697 cm-1
C=O
1695 cm-1
C=O
1569 cm-1
C=C
1079 cm-1
C-O
1146 cm-1
C-O
1563 cm-1
C=C
1362 cm-1
C-O
1237 cm-1
C-O
1151 cm-1
C-O
WSB
WSB + NAP

b)

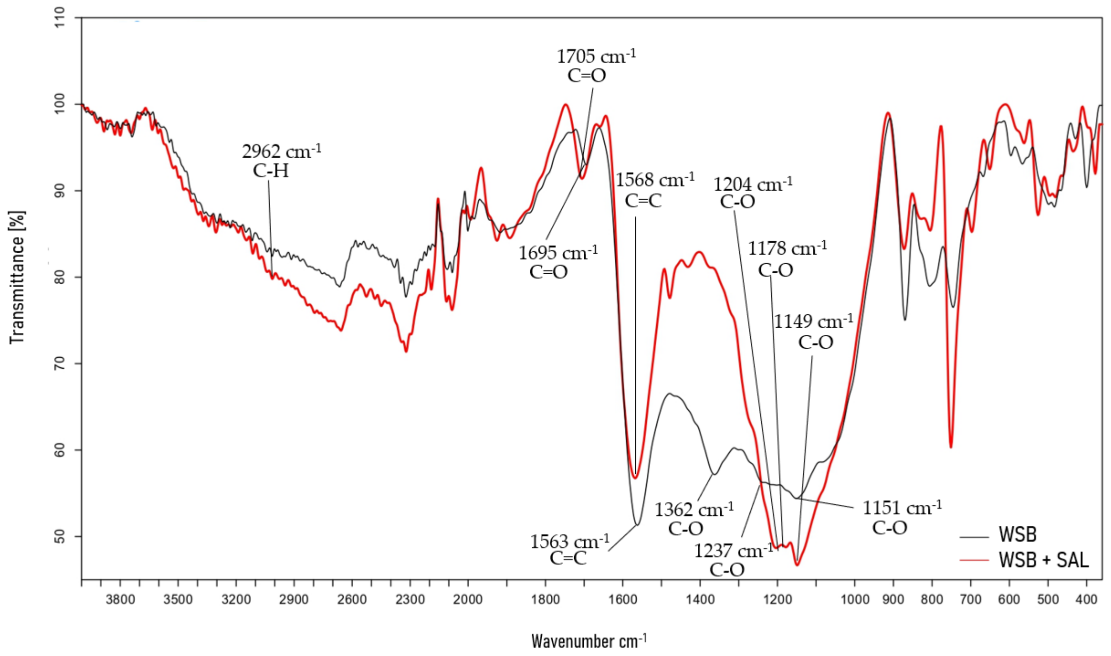
Transmittance [%]
Wavenumber cm-1
2962 cm-1
C-H
1705 cm-1
C=O
1695 cm-1
C=O
1568 cm-1
C=C
1563 cm-1
C=C
1362 cm-1
C-O
1237 cm-1
C-O
1204 cm-1
C-O
1178 cm-1
C-O
1149 cm-1
C-O
1151 cm-1
C-O
WSB
WSB + SAL

c)

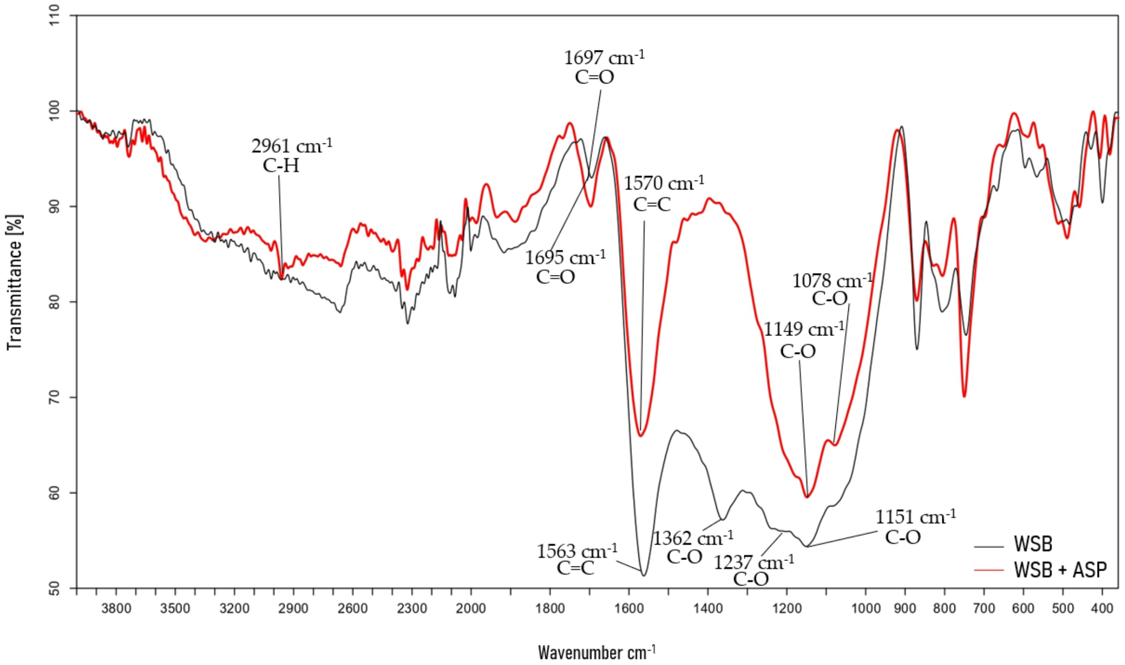

Figure S1. FTIR spectra of WSB before and after a) NAP, b) SAL, and c) ASP adsorption.

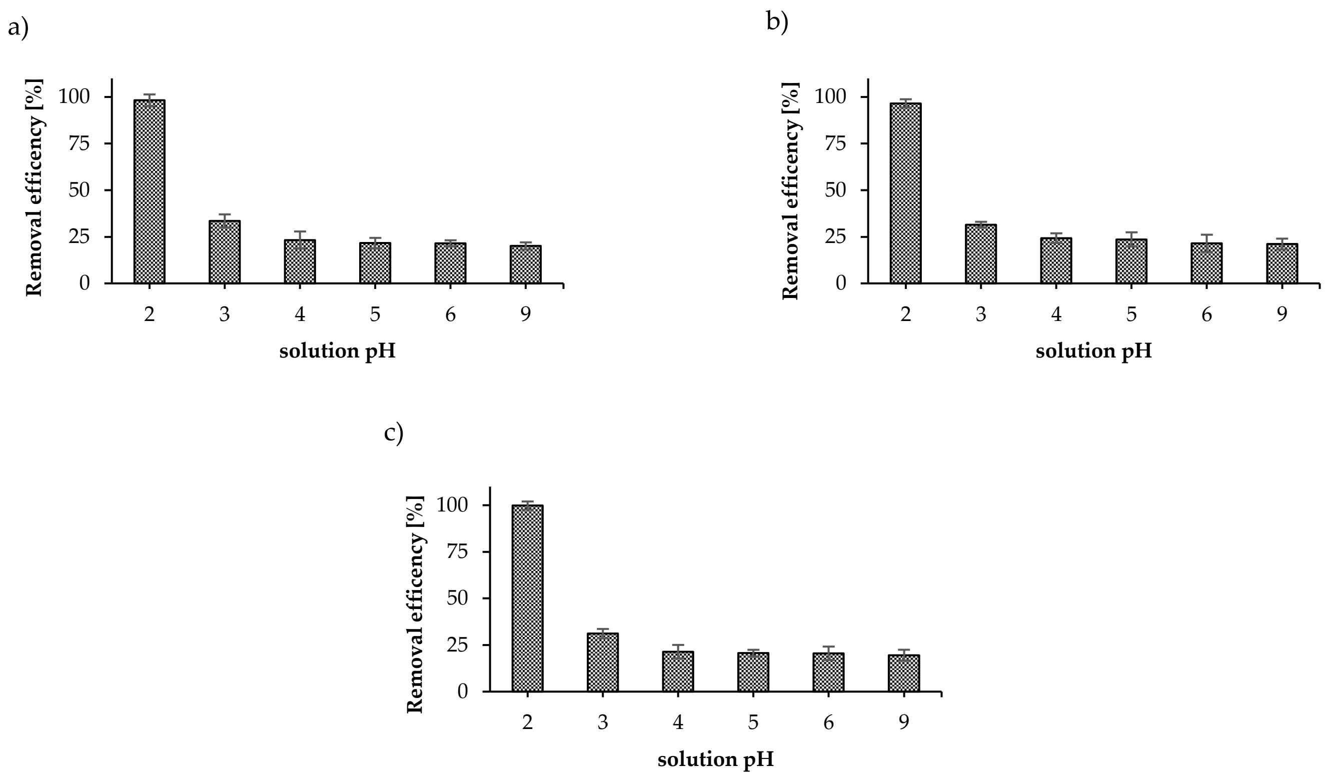

Figure S2. Effect of the solution pH on the removal efficiency of a) SAL, b) ASP, and c) NAP (conditions: contact time 1 h, drug concentration 25 mg/L, room temperature, sorbent dosage 1 g/L).

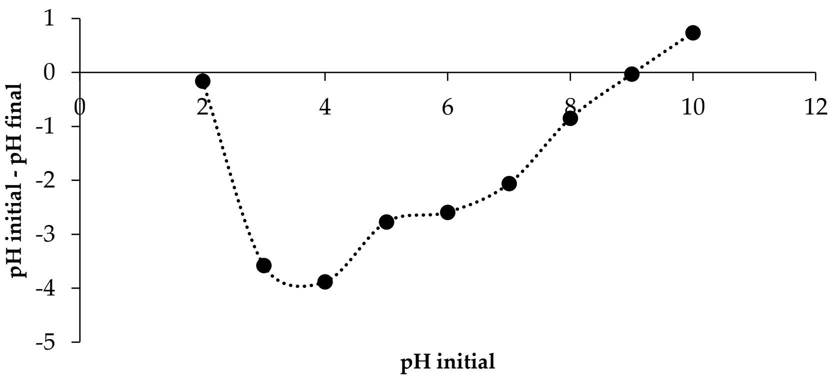

Figure S3. Plot of pH point of zero charge of WSB.

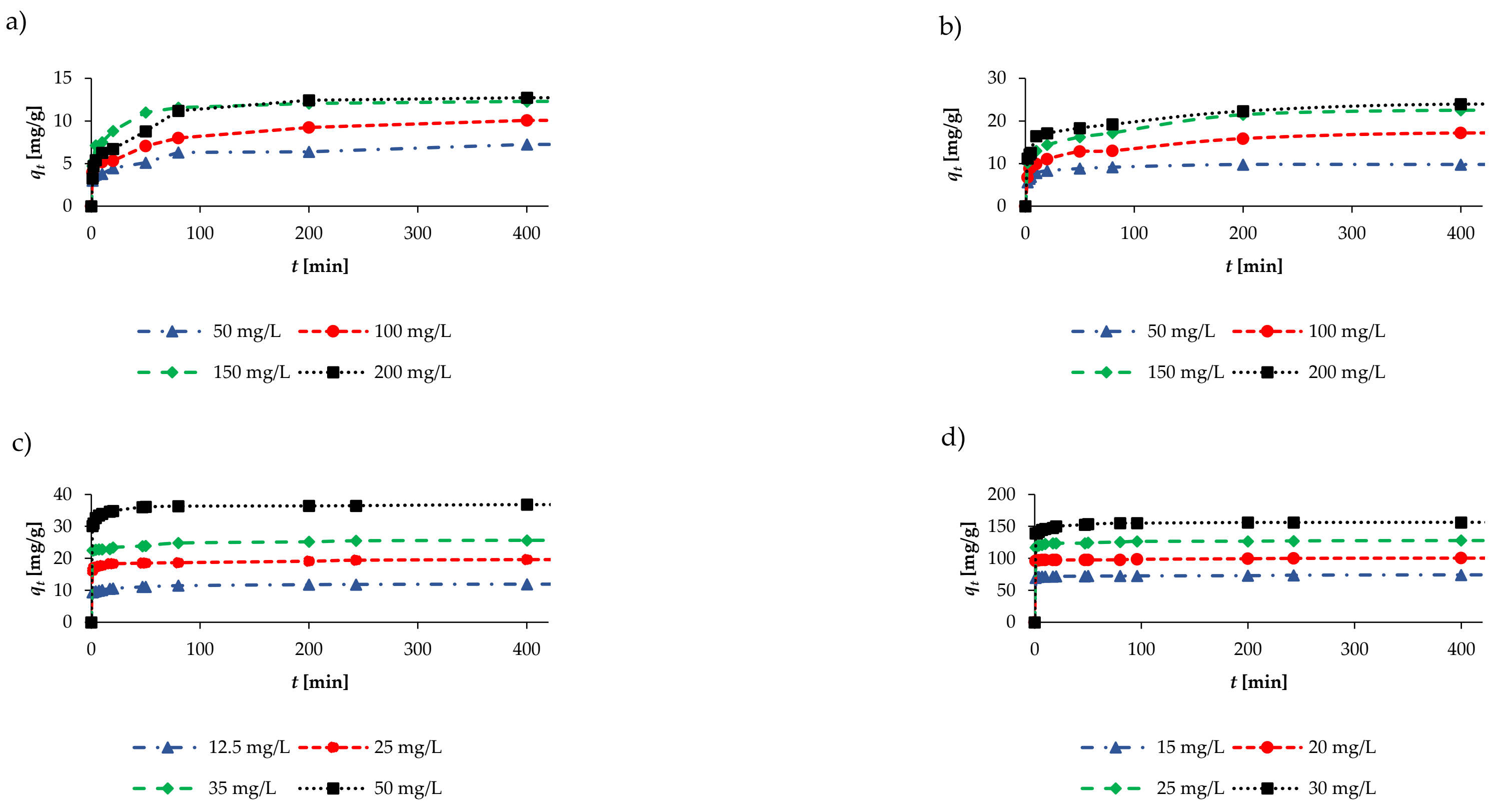

Figure S4. Effect of the contact time on adsorption capacity of a) ASP, b) SAL, c) KET, and d) NAP.

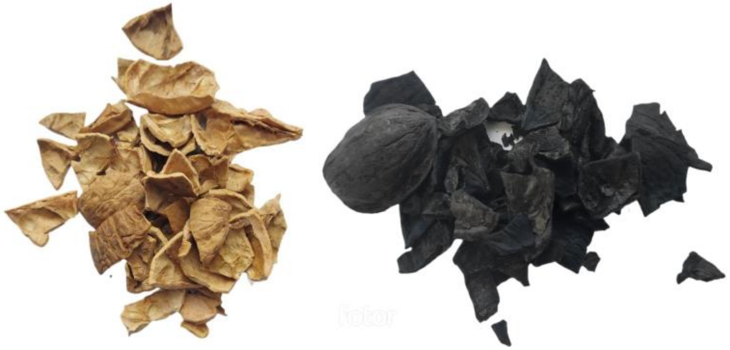

Figure S5. The raw, crushed walnut shells (left) and obtained WSB prior grounding (right).

Table S1. Characterization of tested pharmaceuticals.

Name	Abbreviation	Structure	CAS No.	Molecular weight [g/mol]	pK_a	$\log K_{ow}$
aspirin	ASP	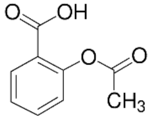	50-78-2	180.16	3.49	1.19
salicylic acid	SAL	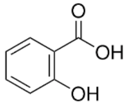	69-72-7	138.12	2.99	2.26
ketoprofen	KET	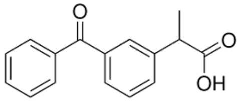	22071-15-4	254.28	4.45	3.12
naproxen	NAP	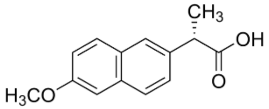	22204-53-1	230.26	4.15	3.18

Table S2. Biosorption kinetic parameters for adsorption of ASP, SAL, NAP, and KET onto WSB.

C_0 [mg/L]	q_{exp} [mg/g]	Pseudo-first-order			Pseudo-second-order			Elovich model		
		q_{cal} [mg/g]	k_1 [1/min]	R^2	q_{cal} [mg/g]	k_2 [g/mg × min]	R^2	α [mg/g × min]	β [g/mg]	R^2
					**ASP**					
50	10.2459	6.6466	0.0023	0.6912	6.1501	0.0371	0.9830	34.9	1.4269	0.9372
100	15.2787	10.5828	0.0021	0.7318	10.9890	0.0193	0.9748	33.6	0.9985	0.9202
150	20.5246	13.6019	0.0016	0.4993	10.5042	0.0190	0.9754	10.2	0.8064	0.9590
200	20.4590	14.9211	0.0021	0.6777	8.6133	0.0189	0.9721	12.9	0.7198	0.9655
					**SAL**					
50	10.3137	3.2307	0.0055	0.5939	9.7466	0.0473	0.9997	3683.8	1.4349	0.9729
100	19.1176	10.4978	0.0046	0.8426	17.1527	0.0069	0.9954	195.143	0.6177	0.9657
150	26.6245	15.2125	0.0039	0.8042	22.7273	0.0063	0.9955	165.5	0.4806	0.9463
200	30.6735	17.4663	0.0028	0.6832	23.9234	0.0062	0.9969	111.3	0.4686	0.9771
					**NAP**					
15	74.2456	4.5541	0.0076	0.5426	74.0741	0.0169	0.9999	5E+51	1.7939	0.9244
20	100.4125	5.3260	0.0090	0.6068	100.0000	0.0133	1.0000	2E+334	1.7867	0.7810
25	127.9365	8.6477	0.0111	0.6783	128.2051	0.0107	1.0000	6E+30	0.5947	0.9674
30	156.4445	12.2293	0.0111	0.6643	156.2500	0.0093	1.0000	1E+18	0.2929	0.9569
					**KET**					
12.5	12.4023	2.0105	0.0018	0.4178	12.2699	0.0197	0.9997	7E+08	2.2952	0.9676
25	19.9600	2.3367	0.0028	0.6269	19.9203	0.0187	1.0000	2E+13	2.0678	0.9684
35	25.8102	2.7146	0.0041	0.7311	25.8398	0.0179	1.0000	8E+12	1.8015	0.9257
50	37.4716	3.4411	0.0025	0.4425	37.3134	0.0134	1.0000	2E+11	1.0581	0.8826
